# Assessing the EU27 Potential to Meet the Nature Restoration Law Targets

**DOI:** 10.1007/s00267-024-02107-9

**Published:** 2025-01-07

**Authors:** Ilaria Perissi

**Affiliations:** 1 Consiglio Nazionale delle Ricerche, Istituto di Geoscienze e Georisorse, Pisa, Italy; 2 Associate Member of the Club of Rome, Winterthur, Switzerland

**Keywords:** Nature Restoration Law, LULUCF, National Energy Climate Plan, Common Agricultural Policy Plans, Semantic scale

## Abstract

The Nature Restoration Law adopted by the European Union in 2024 aims to implement measures to restore at least 20% of its land and sea by 2030 and all ecosystems in need of restoration by 2050, focusing on among others agricultural land, forests, urban, marine, freshwater, and wetlands areas. The goal is to enhance the natural and semi-natural habitats’ role in achieving climate targets and preserving biodiversity. Member States must submit detailed national restoration plans, outlining specific actions and mechanisms for monitoring progress. However, these plans should align with the ongoing Common Agricultural Policy and National Energy and Climate Plans objectives. Using data from European Commission reports and applying a semantic interval scale methodology, this study quantifies each Member State’s ambitions and effectiveness under the National Energy Climate Plans and Common Agricultural Policy and establishes a benchmark for reporting under the Nature Restoration Law. The findings reveal the National Energy Climate Plans’ wide disparities in implementing decarbonization measures, climate change adaptation and the implementation of nature-based solutions. The Common Agricultural Policy Plans exhibit only partial commitment to greening agriculture, yet their alignment with Nature Restoration Law objectives varies. Therefore, timely coordination between the three strategies is crucial to avoid conflicting goals, overlapping efforts, and wasting time and resources, ensuring the success of restoration actions.

## Introduction

In June 2024 the European Union (EU) took a historic step in environmental policy by formally adopting the Nature Restoration Law (NRL) (European Parliament and Council of the European Union 2024) which entered into force on 18 August 2024. This Regulation represents a pivotal shift in the EU’s approach to environmental conservation, moving beyond mere preservation to actively restoring degraded ecosystems across its Member States (MSs). The law mandates the restoration of at least 20% of the EU’s land and sea areas by 2030, with a long-term restoration goal of all ecosystems in need of restoration by 2050. It includes mandates for increasing populations of grassland butterflies, enhancing organic carbon stocks in cropland soils, and expanding high-diversity landscapes in agricultural regions. Additionally, the law ensures urban green spaces, tree canopy cover, and the restoration of drained peatlands. Restoration of marine ecosystems focuses on revitalising seagrass beds and cold water coral reefs, and ensuring sustainable fishing practices through Regulation (EU) No 1380/2013 on Common Fisheries Policy (European Parliament and European Council 2013), while reducing pollution to support biodiversity and carbon storage. For rivers and freshwater ecosystems, efforts include rewilding rivers by removing barriers, restoring floodplains for ecological resilience, and enhancing riparian vegetation to improve water quality. The NRL also seeks to reverse pollinator decline and strengthen diversity, targeting recovery by 2030 and ensuring sustained growth thereafter. A standardised monitoring method will be developed by 2025 to enable consistent data collection across MSs. Wetlands and coastal areas (RESTORE4CS 2023) are targeted through the restoration of wetlands for biodiversity and carbon capture, along with the protection of marshes and salt marshes to combat erosion and improve coastal resilience. Clear targets have been set for restoring habitats in poor condition, listed in Annex I and Annex II of the NRL, with phased goals to put in place restoration measures of 30% by 2030, 60% by 2040, and 90% by 2050. To ensure the successful implementation of these ambitious goals, MSs are required to submit detailed national restoration plans to the European Commission (EC). Since improved environmental protection requires more than just dedicated environmental policy measures (Grohmann and Feindt 2024), (Hering et al. 2023) these plans must outline the specific measures each country will undertake to meet the targets and include mechanisms for monitoring and reporting progress. Additionally, the restoration plans shall explicitly identify synergies with other key EU policy frameworks, including those targeting climate change mitigation, climate adaptation, land degradation neutrality, and disaster prevention, to strategically prioritize restoration measures. In this study, the role of the Common Agricultural Policy (CAP) (Ackrill 2000) and National Energy and Climate Plans (NECPs) (European Parliament and Council of the European Union 2018a) in tracking greenhouse gas emissions, land use, and agricultural practices, which are crucial for monitoring the ongoing nature restoration actions integrated into these plans, have been investigated.

NECPs and CAP Plans are directly influenced by the Effort Sharing Regulation (ESR) (European Parliament and Council of the European Union 2023a) and the Land Use, Land Use Change and Forestry (LULUCF) Regulation (European Parliament and Council of the European Union 2023b), which play pivotal roles in achieving the EU’s climate targets and the implementation of the NRL. The ESR sets binding greenhouse gas emission targets for sectors not covered by the EU Emissions Trading System, such as transportation, buildings, waste, and agriculture. Each MS has an annual target based on its GDP per capita, adjusted to ensure fair contributions toward the EU’s climate goals. ESR impacts NECPs by requiring sector-specific policies and measures that align with these targets, and it influences CAP Plans by encouraging sustainable practices within agriculture to reduce emissions, thereby supporting ESR targets. The LULUCF Regulation mandates MSs to account for emissions and removals from land use, forestry, and related sectors, ensuring that emissions do not exceed removals. This rule requires NECPs to include objectives for land-sector carbon sinks, like reforestation, conservation, and sustainable land management. For the CAP, LULUCF Regulation directly impacts land and forestry components by encouraging climate-friendly practices that maintain carbon stocks and biodiversity. In summary, the NECPs focus on LULUCF which overlaps with the NRL’s goal to enhance carbon sinks and ecosystem resilience; the CAP’s eco-schemes, conditionality standards, and targets for high-diversity landscapes can support the NRL’s biodiversity restoration targets.

In the present study, I first outline a methodology to quantify each Member State’s progress toward the NRL targets. Then, the results of this analysis are presented and discussed in the context of meeting the objectives set by the EC. The study concludes by suggesting areas for further analysis to deepen these findings.

## Methods

### Data Resources

Under the Climate Governance Regulation (European Parliament and Council of the European Union 2018b), EU MSs were required to submit their draft NECPs for 2021–2030 by December 2018, with final versions due by December 2019. The European Commission reviewed these plans, providing recommendations and publishing EU-wide assessments. In 2023, MSs updated their NECPs, with drafts due by June and final versions by December 2023. The Commission assessed these updates, using 27 Staff Working Documents (SWDs) (European Commission 2024), which are the basis of NEPCs analysis. The 27 SWDs offer a comprehensive and extensive set of information in qualitative assessments that can be converted into numerical scales. The scaling informs on the progress of the draft updated NEPCs of each member state in the period 2021–2030. The primary purpose of NECPs is to ensure that each country contributes to the EU’s targets for reducing greenhouse gas emissions, increasing renewable energy use, improving energy efficiency, and enhancing energy security. However, for the scope of our analysis, the investigation focuses on NEPCs’ decarbonization dimension only, which includes the binding target for net greenhouse gas removals under LULUCF Regulation, adaptation measures, nature-based solutions and the assessment of renewable energy production in compliance with LULUCF Regulation. The CAP Strategic Plans for the period 2023–2027 were approved by the European Commission and came into effect on 1 January 2023. Each EU member state has a strategic plan, except Belgium, which has two: one for Flanders and one for Wallonia. These plans combine targeted interventions to address specific needs and achieve EU-wide objectives, contributing to climate action, the protection of natural resources, and the conservation and enhancement of biodiversity. Regarding the assessment of CAP Plans, the study refers to documents available from the dedicated European Commission (EC) web portal (European Parliament and Council of the European Union 2021),(European Commission 2023).

Variations in data collection, reporting standards, and the qualitative nature of adaptation goals introduce challenges to analysing NECPs and CAP Plans. To address these, EC has developed guidelines and metrics to improve the consistency of assessments. Each MS progress is measured against overarching EU targets rather than past performance, enabling comparability despite differences in national profiles. Transparency is further enhanced through annual progress reports for NECPs and a five-year report for CAP, ensuring ongoing alignment with EU objectives.

For more qualitative aspects like climate adaptation, the EC uses criteria such as resilience, innovation, and risk management to assess MSs’ efforts, focusing on integrating resilience measures rather than numerical targets. Additionally, the European Court of Auditors (ECA) assures consistency and transparency by auditing fund efficiency, data reliability, and the EC’s evaluations. The ECA’s recommendations are received by the EC, helping refine guidelines for future evaluations.

Although the EC sets requirements for NECPs and CAP Plans, MSs retain the flexibility to tailor specific measures based on their unique contexts. For instance, each MS can choose agricultural emission reduction methods, such as methane management in livestock-focused regions or soil carbon practices in areas with extensive cropland. Similarly, each country decides how to better promote carbon sequestration under LULUCF, selecting forestry or soil management practices suited to local landscapes, and prioritizes renewable energy resources, like wind, solar, or bioenergy, depending on MSs climate.

There also can be an overlap between NECPs and CAP, particularly in land use and biodiversity preservation. For instance, NECPs’ LULUCF goals can include actions similar to the agri-environmental or Eco-schemes from CAP, which may raise concerns of double-counting if they are assessed in both plans. However, the EC’s guidelines seek to coordinate reporting requirements to avoid the occurrence of duplicative assessments.

Overall, the author recognises that while the current EC reporting framework for NEPCs and CAP Plans offers a reliable basis for a first assessment of EU27 potential to meet the NRL targets, continuous data updating/refinements are needed to capture the nuanced progress of MSs in achieving environmental goals.

### Scoring Methodology

Due to the qualitative nature of the data collected from the EC documents, I use a semantic interval scale to assess the ambition of objectives, targets and contributions and adequacy of supporting policies and measures of the MSs. A semantic scale (Watanabe 2021) rates an adjective or concept and is particularly effective in expressing intensity levels.

#### NECPs assessment

The analysis of the EC on the effectiveness of NECPs is conducted using qualitative evaluations which have been retrieved from the SWDs assessments and summarised in tables as detailed in the supplementary material Excel files.

The EC assessment of the Draft updated NECPs report 5 main dimensions: Greenhouse Gas Emissions Reductions, Increased Renewable Energy Use, Energy Efficiency Improvements, and Energy Security Competitiveness of European Industry. Each dimension has subtasks and objectives. The decarbonization dimension, which the present analysis focuses on, presents the EC assessment for the following task:

1. Greenhouse gas emissions, removals and storage
  1. Commitment to achieve climate neutrality by 2050
  2. Ambition under ESR
  3. Ambition of the LULUCF Regulation
  4. Measures for LULUCF to reach the targets
  5. Circular economy
  6. Mobility
  7. Assessment of the impact of policies and measures on the achievement of the GHG mitigation targets
  8. Carbon Capture Usage Storage (CCUS)
  9. National target and projection for agriculture
  10. Mitigating non-CO2 emissions
  11. Targets and international commitments under the Paris Agreement
  12. Long Term Strategy
2. Adaptation
  13. Adaptation goals and targets
  14. Nature-based solutions
3. Renewable energy
  15. Compatibility with LULUCF Regulation

For renewable energy, the decarbonization analysis specifically examines the production involving land use, such as biomass.

Using a numerical interpretation of the EC assessment helps create graphical representations of the EU27’s individual and collective efforts towards decarbonization goals related to LULUCF, adaptation, and renewable energy production. This approach also aids in visualizing MSs natural restoration efforts. Similar to the previous assessment of NECPs by Perissi and Jones (Perissi and Jones 2022), the author converted qualitative comments for each task into “scores” based on a “semantic scale” principle. Unlike the previous EC SWDs (SWD/2020) (European Commission 2024), the EC observations of the SWD (2023) are not summarized in an “evaluation table” but are detailed in each SWD section. However, converting these observations into a synthetic scored evaluation (Table 1) is straightforward as most of the observations include the evaluation itself.

**Table 1:** Semantic scale evaluations assigned by the author to score the EC Assessment of the Member States updated NEPCs

Evaluation	Score
Fully	5
Largely	4
Sufficiently	3
Partially	2
Low	1
Not addressed (N/A)	0

#### CAP Plans assessment

Regarding the assessment of CAP Plans, the investigation considers the “CAP Plans by Country” available on the EC webpage under Agriculture and Rural Development (European Parliament and Council of the European Union 2021). These plans provide an overview from 2023–2027, focused on social, environmental and economic goals. A first assessment of these plans by the EC is present in the same online source (European Commission 2023). This report’s analysis revolves around evaluating the joint effort, common ambition, and potential impacts of the CAP Strategic Plans focusing on modernizing agriculture through innovation, digitalisation, and knowledge sharing. It examines the design, financial allocations, and expected outcomes of these interventions, offering a comprehensive analysis of their potential impact. However, the author proposes here a structured assessment approach based on scoring that can provide a more detailed, comparative, and objective assessment of the CAP Strategic Plans, constituting a benchmark in future reporting for the NRL and the CAP Plans themselves, identifying more effective and targeted interventions across the EU.

The analysis focuses on plans’ potential of being greener and less greenhouse gas intensive than in the past, exploring the main 6 dimensions committed by the CAP to achieve these greener plans:

1. Higher Green Ambitions
2. Contribution to Green Deal Targets
3. Enhanced Conditionality
4. Eco-Schemes
5. Rural Development
6. Climate and Biodiversity

The scoring criteria are the same as the NEPCs evaluation (Table 1). Details of the evaluation are reported in the supplementary materials.

## Results

The results of the study are organized into 2 parts: results on NECPs assessment and results on CAP Plans assessment.

### Assessment of NECPs decarbonization targets, ambition and measures

NECPs results are in turn split into 3 parts: GHG emissions removals and storage, Adaptation and Renewable energy

#### GHG emissions, removals and storage

This section concerns the analysis of the first 12 points listed in Methodology 2.2. These 12 points have been arranged into 5 groups that highlight 5 different areas of the plans:

1. Target and commitments to net zero society: this section points out the MS commitment to a Net Zero society in terms of clarity of plans in describing the country’s targets for Net Zero 2050:
  Commitment to achieve climate neutrality by 2050
  Targets and international commitments under the Paris Agreement
  Long Term Strategy
2. Target and Ambition 2030: This section points out the MS commitment to the intermediate goals and targets by 2030 with a focus on ESR and LULUCF Regulations’ objectives.
  Ambition under ESR (MSs obligation for the ESR 2030)
  Ambition of the LULUCF Regulation (MS obligation by 2030)
3. Measures focused on Land Use and Agriculture: this part establishes the measures to achieve the target and their adequacy to the country’s ambition with a focus on Land Use and Agriculture
  Measures for LULUCF to reach the targets
  National target and projection for agriculture mitigating non-CO2 emissions
4. Other Measures: reports on other decarbonisation measures ongoing in different sectors than LULUCF
  Policies and measures for circular economy
  Policies and measures for improved access to zero mobility
  Deployment of Carbon Capture, Use and Storage (CCUS)
5. Measures based on an analytical basis: this part assesses the impact of policies and measures on the achievement of the GHG mitigation targets using an analytical basis

The author has converted the previous areas’ qualitative assessments from the SWDs in scores, according to Table 1. The total score per area for each MS is obtained by summing the scores of the points within each area (detailed in the supplementary material).

**Fig. 1:**
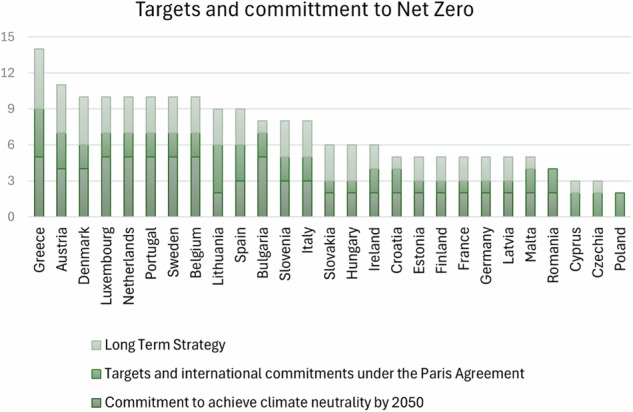
Classification of MSs on their quantitative NECPs assessment based on evaluation scores for targets and commitments to net zero society

Figure 1 reports the classification of MSs according to their scores in the different areas. In their NEPCs, 13 MSs have addressed the level of commitment and targets to Net Zero society between partially and sufficiently; only 3MSs have not or very low reported about commitment for Net Zero.

Sweden aims for climate neutrality by 2045, while Germany has also set a 2045 target but lacks concrete pathways for 2030 and 2050. Fully committed countries, such as Bulgaria, Belgium, Greece, Hungary, Ireland, and Lithuania, have clear plans that align with the 2050 target. However, some countries have only partially addressed this commitment. For example, Croatia and Estonia acknowledge the 2050 goal but do not provide specific strategies or concrete pathways to achieve it. Others, such as Cyprus and Czechia, do not address the goal at all, highlighting a lack of strategic direction and ambition in their national plans. A few countries, including Denmark and Finland, aim to bring forward their climate neutrality targets, but their plans still lack comprehensive strategies for reaching the 2050 goal.

Most countries’ strategies under the Paris Agreement (Perissi et al. 2018) show partial or limited progress. Portugal and Spain stand out, having fully embedded the increased targets of the ESR and the LULUCF Regulation. Many countries, such as Belgium, Croatia, Czechia, and Malta, only partially address these increased targets and lack concrete plans to phase out fossil fuel subsidies (Drake and Skovgaard 2024). France, Germany, and Estonia demonstrate low or limited commitment to their national plans toward the Paris Agreement. These plans fail to reflect comprehensive progress toward international obligations, particularly concerning eliminating fossil fuel subsidies. This need, however, is only starting to be recognised in United Nations climate change negotiations (van Asselt et al. 2024). Latvia does not address this aspect, indicating significant room for improvement in aligning national policies with global climate targets.

Long-term strategies to reach climate neutrality by 2050 vary significantly across the EU27 countries (Bluszcz et al. 2024),(Perissi and Jones 2024) despite the clear framework of mitigation policies (Wang et al. 2024). While some, like Greece, Luxembourg, Portugal, and Romania, have fully addressed this aspect by including concrete pathways for 2030, 2040, and 2050, others have fallen short. Belgium has sufficiently addressed its long-term strategy, providing a pathway to 2030 and some variables extending to 2050. Several countries, such as Estonia, Finland, France, and Germany, partially address long-term strategy by mentioning targets but lacking detailed pathways and assessments. On the other hand, nations like Bulgaria, Croatia, Malta, and Poland need significant improvement, as their plans do not include concrete pathways to 2050.

**Fig. 2:**
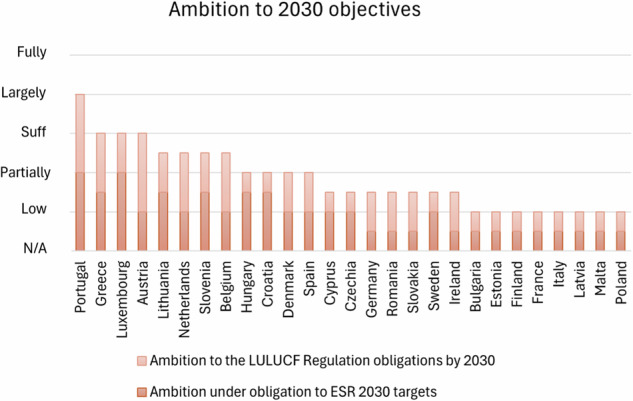
Classification of MSs on their quantitative NECPs assessment based on evaluations scores illustrating MSs ambition levels toward 2030 objectives

MSs ambition to the 2030 intermediate objectives is only sufficiently addressed by 3 MSs, while MSs did this partially (Fig. 2).

Several countries have adopted more ambitious national targets than those required by the ESR, but there are clear disparities in their approaches and readiness to meet the 2030 targets. Notably, countries such as Croatia, Greece, Hungary, Lithuania, Luxembourg, Portugal, Slovenia, Spain, and Sweden have been rated as sufficiently addressed or largely indicating a higher level of ambition or progress. Spain and Sweden stand out, with Spain’s plan setting a more ambitious national target than the ESR requirement and Sweden’s plan aiming for significant reductions, though both lack detailed projections under additional measures. Bulgaria, Estonia, Finland, France, Germany, Ireland, and Italy, have been identified with low ambition. These countries either lack sufficient policies to meet their targets or fail to provide adequate projections. This highlights a need for more robust policy frameworks and implementation to ensure these nations meet the ESR obligations by 2030. Belgium, Cyprus, and Czechia have set more ambitious national targets than the ESR, but they lack additional projections, which limits the credibility and potential success of these targets. A recent study by Ricciolini et al. (Ricciolini et al. 2024) highlights countries’ disparity in achieving 2030 targets relies on a weakened implementation of the EU’s Cohesion Policy (Amendolagine et al. 2024).

Assessment of LULUCF ambitions similarly presents a mixed picture as recently investigated by Di Lallo et al. (Di Lallo et al. 2024). Belgium, Greece, Luxembourg, the Netherlands, and Portugal are among the few countries whose plans sufficiently increased the ambition of the LULUCF Regulation and included a pathway toward achieving national targets with a commitment to enhancing carbon sinks (Mabidi et al. 2024). However, the majority of countries fall short in this: Bulgaria, Croatia, Cyprus, Czechia, Estonia, Finland, France, Germany, Hungary, Ireland, Italy, Latvia, Lithuania, Malta, Poland, Romania, Slovakia, Slovenia, Spain, and Sweden have plans that either do not reflect the increased ambition of the LULUCF Regulation or lack a clear pathway towards their national targets. This widespread lack represents a major gap in the overall EU effort to leverage land use and forestry as a means to offset emissions.

**Fig. 3:**
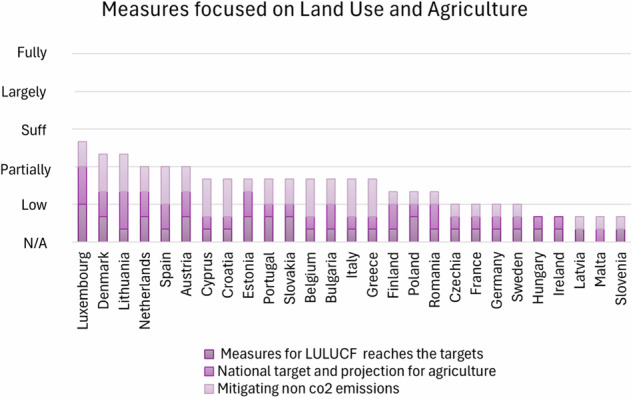
Classification of MSs on their quantitative NECPs assessment based on evaluations scores: measures from Land use and Agriculture

**Fig. 4:**
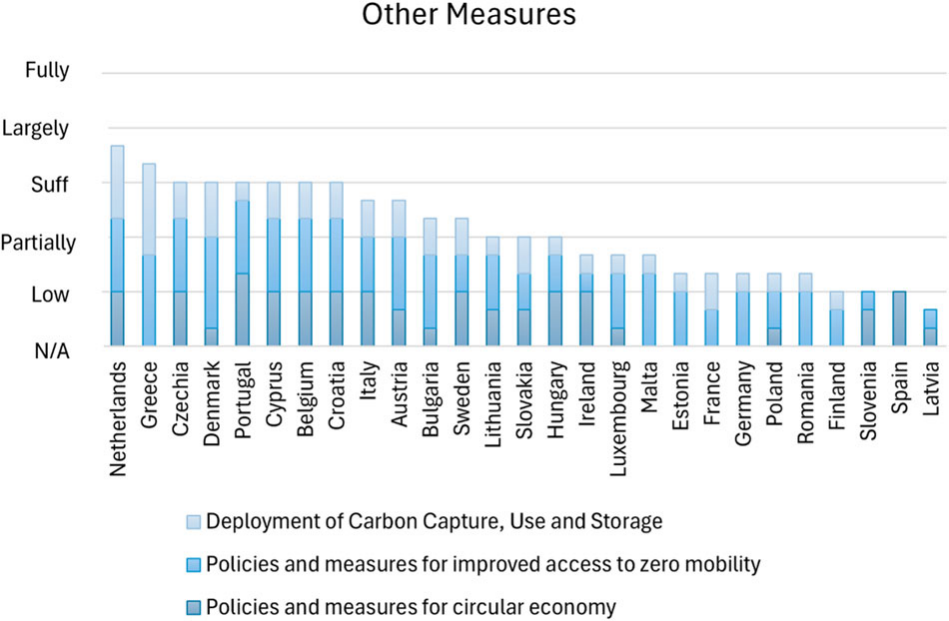
Classification of MSs on their quantitative NECPs assessment based on evaluations scores: measures from other decarbonization actions

Regarding the MSs plans assessment of measures, any of the MSs sufficiently describes measures to reduce emissions in the Land Use and Agriculture sectors (Fig. 3). In comparison to Perissi and Jones’s previous NEPCs assessment in 2022 (Perissi and Jones 2022), most MSs progressed mainly with measures to access net zero mobility first (Fig. 4).

In terms of measures for LULUCF to reach the targets, many EU countries need to strengthen their strategies. Countries such as Bulgaria, Belgium, Croatia, Cyprus, Czechia, Finland, France, Germany, Greece, Hungary, Italy, Lithuania (Dagiliūtė and Kazanavičiūtė 2024), Malta, Poland, Romania, Slovakia, Slovenia, Spain, and Sweden, often list policies but lack concrete details on their implementation, impact quantification, and monitoring, resulting in unclear pathways to achieve the 2030 LULUCF targets (Luo et al. 2024).

Addressing agricultural emissions remains a significant concern (Kamyab et al. 2024). Most countries lack detailed pathways and quantifications for reducing agricultural emissions. Belgium, Croatia, Cyprus, Czechia, Finland, France, Germany, Greece, Hungary, Italy, and Ireland show marginal decarbonization actions. Latvia, Lithuania, Luxembourg, Malta, the Netherlands, and Poland have not addressed agricultural emissions in their plans, highlighting a lack of clear targets and strategies in this sector. Only a few countries, including Denmark, Estonia, Portugal, Romania, Slovakia, Slovenia, and Spain, have made partial progress in outlining plans to meet national agricultural emission targets.

To mitigate non-CO_2_ emissions, there is a wide variance in how countries are addressing this issue. Belgium, Croatia, Cyprus, Denmark, and Greece have addressed non-CO_2_ emissions, incorporating plans to tackle methane and fluorinated gases (F-gases). However, many other countries exhibit significant gaps. Estonia, Finland, France, Germany, Hungary, Ireland, Italy, Latvia, and Lithuania lack detailed projections and specific measures to manage non-CO_2_ emissions. Other countries have only partially addressed this issue, as their plans cover some sectors but often lack strategies for methane from enteric fermentation and F-gases. A study by Shindell et al. (Shindell et al. 2024) shows that mitigation cost non-CO_2_ emissions are generally low when compared to real-world financial instruments and significantly lower than their estimated damages. However, to promote the adoption of even cost-saving measures, it is essential to implement legally binding regulations and widespread pricing mechanisms.

For circular economy policies, Belgium, Croatia, Cyprus, Czechia, Italy, and Portugal planned specific policies and measures promoting a circular economy, while many others, including France, Germany, and Spain, lack specific policies or the recognition of circular economy practices as a tool for decarbonization. The adoption of circularity measures and the incorporation of European directives related to the circular economy are still in the early stages though (Galdeano-Gómez and García-Fernández 2024).

Zero-emission mobility is better integrated across the EU27. Most of the countries include initiatives like electrification of transport, public transit infrastructure improvements, and alternative fuel networks. Effective coordination with stakeholders and new governance structures is crucial. In general, cities with strong research and innovation backgrounds are better prepared (Christidis et al. 2024).

CCUS adoption across MSs is mixed. Greece significantly planned CCUS. Most countries’ plans typically include information on CO_2_ capture and storage but lack details on specific projects, targets, or comprehensive deployment strategies. In Germany, Finland and Slovenia CCUS is prohibited. Nevertheless, in a world scenario, CCUS technologies in Europe are deeply investigated and among the most advanced (Chu et al. 2024).

**Fig. 5:**
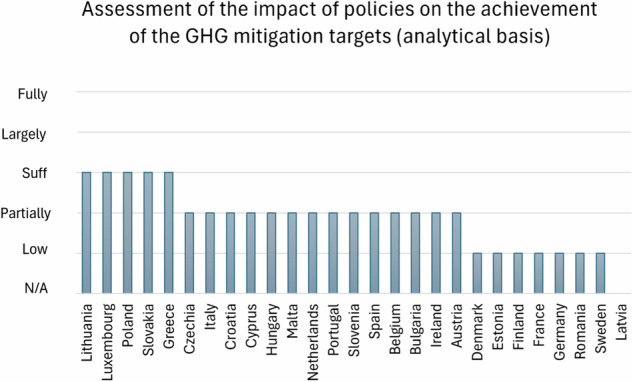
Classification of MSs on their quantitative NECPs assessment based on evaluations scores: assessment of policies and measures based on an analytical basis

Despite the variety of the previously analysed decarbonization measures, most (13 MSs) are only partially developed on an analytical basis: 5 MSs furnish sufficient implementation of the measures, and 9 MSs are low or N/A (Fig. 5).

Countries such as Bulgaria, Belgium, Croatia, Cyprus, Czechia, and Sweden included some form of impact assessment in their plans but fell short of providing detailed quantification of the effects of individual policies across all relevant sectors. This lack of granularity means that while some broad assessments are present, the specific impacts on GHG emissions remain unclear, potentially undermining the reliability of their mitigation strategies. Denmark, Estonia, Finland, France, and Germany provide only partial or qualitative evaluations of their policies’ impacts on GHG emissions, often with insufficient detail to ensure that their targets will be met. The assessments are either brief or deferred, lacking the necessary depth to fully understand the effectiveness of their climate actions.

On a more positive note, Greece’s plan includes a detailed assessment of the impacts of its policies, although it still lacks comprehensive detail. Luxembourg stands out with a thorough assessment, incorporating detailed evaluations of how its policies and measures will impact GHG emissions, thus ensuring a robust framework for meeting its targets across all sectors.

The disparity in the quality of impact assessments across EU-27 countries indicates a broader issue of varying levels of readiness and rigour in climate planning. While some countries have developed detailed and comprehensive evaluations, many others have only partially addressed or inadequately assessed the impacts of their policies. This inconsistency underscores the need for more detailed and uniform approaches to policy impact assessment, which are crucial for effectively tracking progress and ensuring the successful attainment of GHG reduction targets (Fujiwara et al. 2019).

**Fig. 6:**
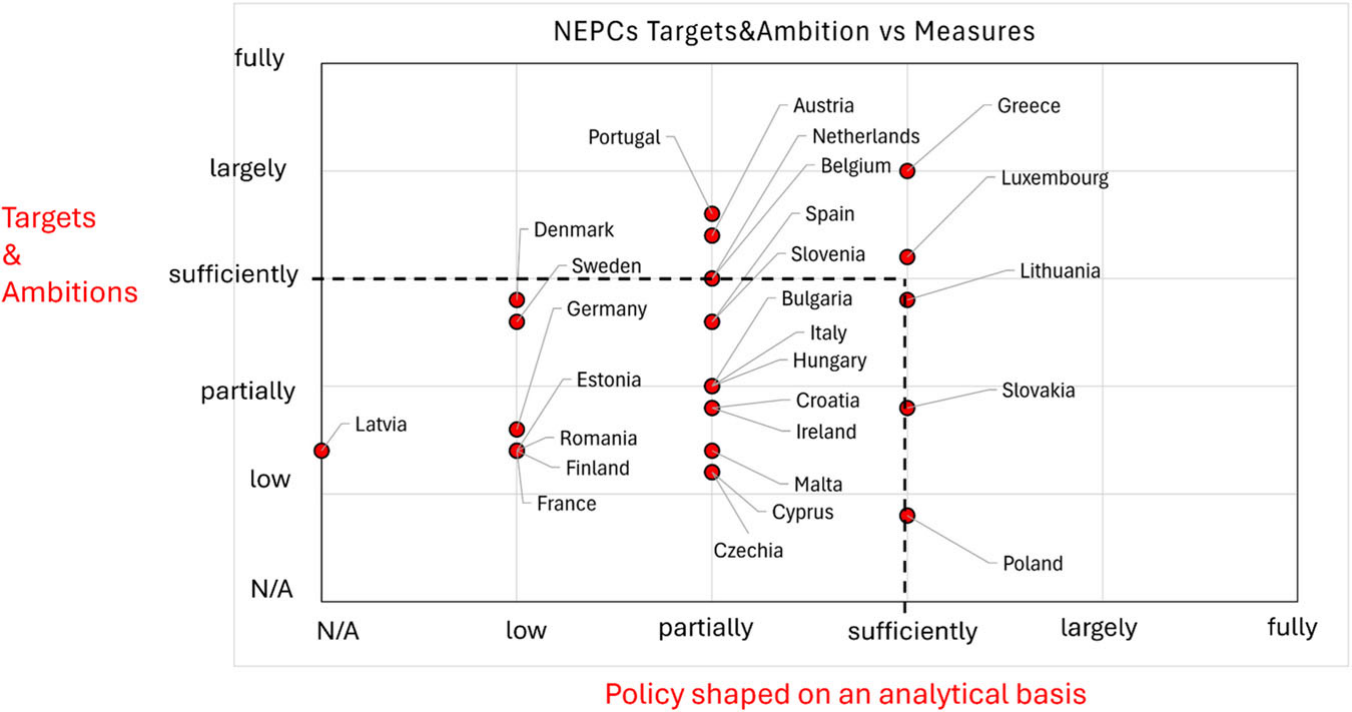
Mapping EU27 MSs adequacy and policy details to achieve Net Zero by 2050 based on EC SWDs 2023. LULUCF and Agriculture sectors are among the weaker sectors in the deployment of impactful decarbonization policy (Classification is obtained by summing scores from Targets plus Ambitions vs scores obtained from Policy shaped on an analytical basis)

Figure 6 maps the EU27 distributions of policy strength: just 7 MSs can account for an ongoing adequate (from sufficient to higher) policy implementation of measures based on an analytical basis and concerning the set targets.

In summary, NECPs decarbonisation strategy is still unbalanced toward setting clear targets and ambitions while revealing weaknesses in implementing strong actions to achieve them. The credibility of MS’s policy is, on average, not yet strong enough to grant the Green Deal goals achievement, especially in implementing decarbonization actions within the LULUCF sector.

#### Adaptation

**Fig. 7:**
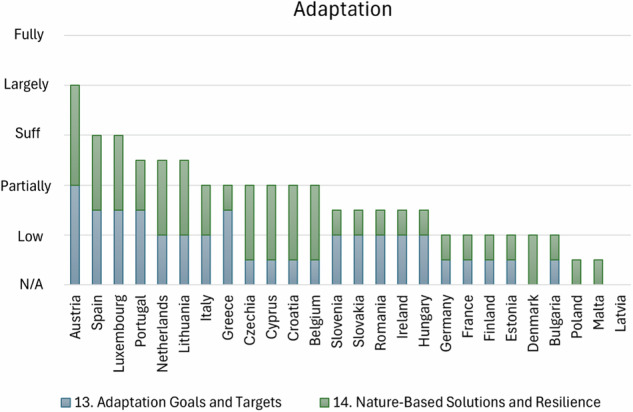
Classification of MSs on their quantitative NECPs assessment based on evaluation scores evaluating MSs adaptation assessment

The EU policy on climate adaptation prioritizes urgent action in ecosystems, health, and food, indicating these areas require immediate interventions to build resilience against climate impacts (European Energy Agency 2024). However, the present assessment of MSs adaptation goals and targets reveals significant variability in how countries approach climate adaptation (Fig. 7). A common issue is the lack of specific goals and measurable targets, even in plans where adaptation needs are acknowledged. This lack of specificity undermines the ability to track progress or implement targeted measures, leaving adaptation efforts vague and difficult to evaluate (Malik and Ford 2024). Many plans also suffer from incomplete vulnerability assessments. While climate risks are generally acknowledged, there is often insufficient analysis of specific vulnerabilities in sectors like energy and water. To fill these gaps, a holistic approach is needed: while vulnerability, adaptation, and resilience are separate concepts, they are deeply interconnected and inherently related. By adopting a framework that takes into account the relationships between social, economic, and environmental factors, stakeholders can formulate more efficient and fair adaptation strategies (Zhai and Lee 2024).

In general, many countries recognize the importance of nature-based solutions but fall short in detailing specific measures, planned investments, or quantifying their impacts on climate resilience (Key et al. 2022). Countries with more comprehensive plans, where nature-based solutions are sufficiently addressed show a commitment to using natural systems to enhance resilience. These plans often include specific actions that focus on integrating nature-based solutions in sectors like water management, forestry, and agriculture. On the other hand, many plans lack quantification of the expected impacts or investments in nature-based solutions, which diminishes the potential of these solutions in mitigating climate risks (Gopinadh Garre 2024). However, in some cases, nature-based solutions are either minimally mentioned or omitted entirely, showing a lack of prioritization for these important tools.

#### Renewable energies

**Fig. 8:**
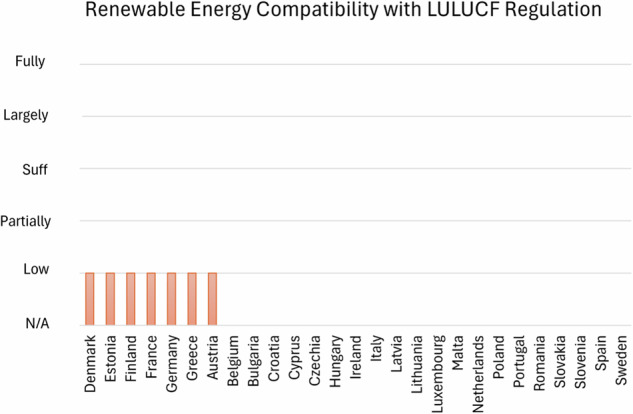
Classification of MSs on their quantitative NECPs assessment based on evaluation scores evaluating MSs Renewable energy compatibility with LULUCF Regulation

The review of EU countries’ NECPs regarding renewable energy compatibility with LULUCF Regulations shows a common gap in comprehensive assessments (Fig. 8). Across most countries, there is a noticeable absence of detailed evaluation on how renewable energy targets interact with LULUCF Regulations, especially regarding sustainability criteria and potential impacts on carbon sinks and land use. However, this is not completely unexpected due to the ongoing challenges in integrating renewable energy with land use strategies, especially concerning the LULUCF framework (Eitan 2024), (de Boer et al. 2015).

Many plans fail to consider how expanding renewable energy infrastructure, such as bioenergy or solar farms, might affect land-use patterns, biodiversity, and carbon sequestration. This omission could lead to unintended consequences where renewable energy targets undermine LULUCF goals, particularly in the forestry and agriculture sectors. Several countries, including Bulgaria, Belgium, Croatia, Cyprus, and others, do not address this compatibility at all, pointing out a critical gap in their plans. In these cases, renewable energy targets are set without accounting for their potential impacts on land-use strategies. Similarly, low levels of assessment are seen in countries like Denmark, Estonia, Finland, and Germany, where renewable energy goals are mentioned but without sufficient detail on their alignment with LULUCF Regulations.

### CAP Strategy Plans mitigation measures

The CAP for 2023–2027 includes several different areas aimed at making the plans greener and more sustainable. Here are some key points:

1. Higher Green Ambitions: each EU country must display a higher ambition for environmental and climate action compared to the previous programming period. This means no “backsliding” is allowed.
2. Contribution to Green Deal Targets: the national CAP Strategic Plans must contribute to the Green Deal targets, including those set out in the Farm to Fork Strategy and the EU Biodiversity Strategy for 2030.
3. Enhanced Conditionality: beneficiaries of the CAP have their payments linked to a stronger set of mandatory requirements. This includes adhering to key EU laws on climate change, energy, water, air, biodiversity, and pesticides.
4. Eco-Schemes: at least 25% of direct payments must be allocated to eco-schemes, which finance environmentally and climate-friendly practices.
5. Rural Development Budget: at least 35% of the total rural development budget must be spent on interventions relevant to the environment and climate, or animal welfare.
6. Climate and Biodiversity: 40% of the CAP budget has to be climate-relevant and strongly support the general commitment to dedicate 10% of the EU budget to biodiversity objectives

**Fig. 9:**
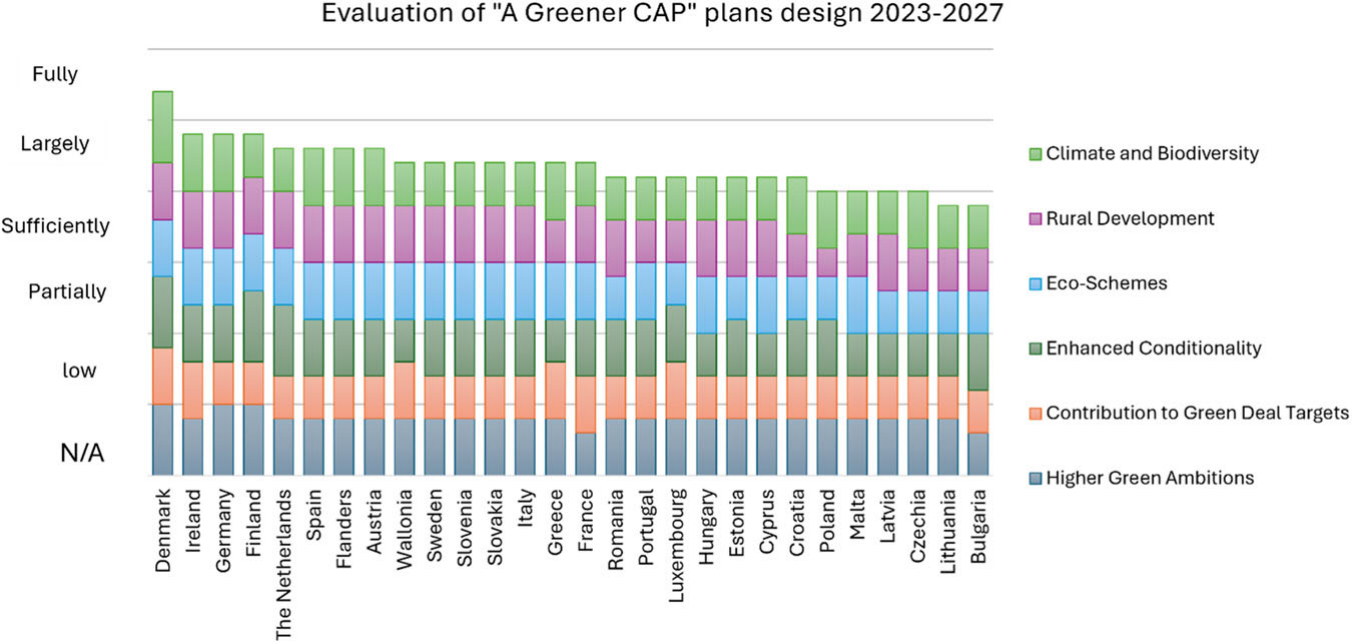
CAP Plans design evaluation with respect to the previous cycle. All MSs show that they include, at least sufficiently, the six key points of “A greener CAP”

This analysis focuses on the previous 6 points as they are strictly connected with land use management and climate change. According to the scoring methodology, each dimension for each MS has been evaluated. Evaluations are related to the 28 CAP Strategic Plans (one for each EU country and two for Belgium) approved by the Commission at the end of 2022 and marking the start of the new Common Agricultural Policy, scheduled on 1 January 2023. The results are summarized in Fig. 9.

The analysis results show all EU MSs have submitted agricultural plans whose design has been improved toward the new environmental objectives set by the EU in comparison to the past period 2014–2022. This is in agreement with a recent evaluation of the plans by the European Court of Auditors (ECA): “the new green architecture enables a higher level of ambition” even though ECA also remark “However, the achievements depend on how member states translate green architecture into their Plans” (European Court of Auditors 2024). According to ECA the plans mainly lack measurable criteria even if they show a “greener architecture”. The author highlights the ECA audit covered the period from June 2018 until April 2024: since the Green Deal only came into effect in 2022, its objectives are likely to encounter a challenging reception within the framework of the CAP Plans. The actual impact of the present plans will be assessed more accurately starting from the end of 2024 when the first reporting period of MSs is due.

MSs will present an annual performance report and hold an annual review meeting with the Commission, that will undertake a first performance review of each CAP Strategic Plan and request, if necessary, specific follow-up actions to EU countries. However, the recommendations from the ECA audit directed toward the EC remain highly significant to frame the review dialogue that will be initiated by EC with each MS from the end of 2024. To identify those actions that will most effectively contribute to or conflict with the implementation of the Nature Restoration Law, a comparative analysis of MSs CAP Plans proposals is necessary to set a baseline for the NRL reporting that ensures that economic, environmental and human resources are not wasted, avoiding overlapping of efforts across the 2 plans.

**Fig. 10:**
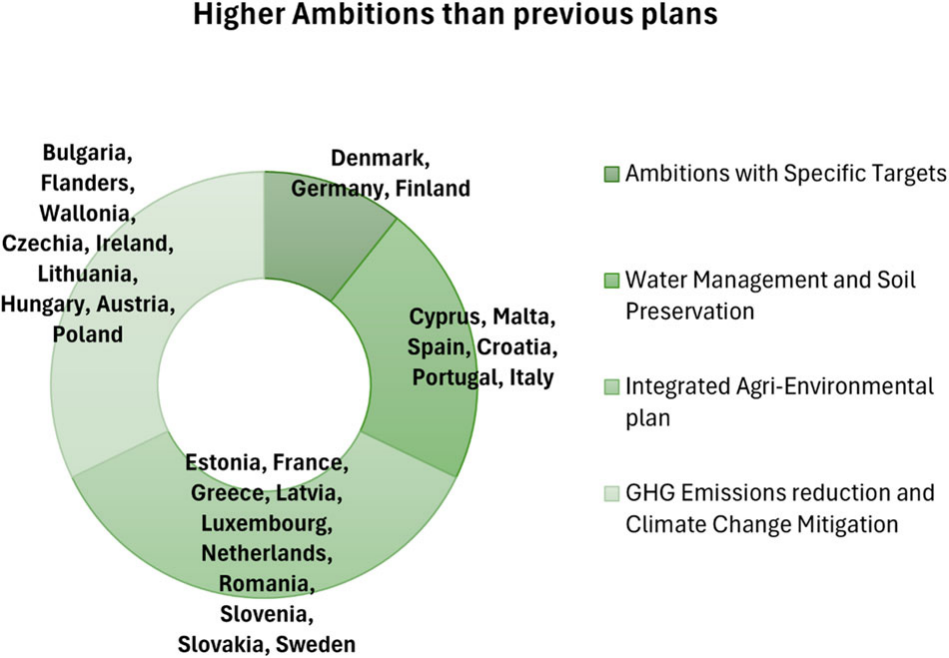
Greener CAP Ambitions priorities of MSs identified by the author

For this reason, for each of the 6 dimensions of the “Greener Cap” objectives, sub-areas that the MSs may have in common were identified by the author. The grouping was based on thematic similarities, such as shared environmental goals, financial allocations, or policy measures. The subcategories were informed by recurring patterns, the scope of MSs’ ambitions, and their reported actions, which I then mapped to specific countries or regions for comparative insights. In this way, I can assess which areas of action are widely spread, while others are less, likely because more difficult to implement. For instance, concerning point 1 (Higher Green Ambitions than previous plans) 4 sub-areas in the MSs were identified according to the MSs priorities (Fig. 10).

Figure 10 shows MSs exhibit a diverse range of environmental and climate ambitions. Denmark, Germany, and Finland demonstrate high ambitions with specific targets (setting a numerical target), such as significant reductions in greenhouse gas emissions and or achieving carbon neutrality by 2050 or earlier. Countries like Cyprus (Sofroniou and Bishop 2014), Malta, Croatia, Portugal, Italy and Spain focus on water management and soil preservation (Mäkipää et al. 2024), addressing issues like desertification prevention, erosion control, and measures to protect soil carbon content and biodiversity, while addressing the impacts of forest fires, water pollution, and emissions from agriculture. Estonia, France, and Greece prioritize an “Integrated Agri-environmental plan”, allocating substantial resources to carbon sequestration and protection of valuable grasslands and ecosystems. Meanwhile, Bulgaria, Flanders, and Wallonia align their efforts with the Green Deal ambitions, focusing on greenhouse gas emissions and climate change mitigation. Each country’s approach highlights a commitment to addressing environmental challenges through tailored strategies that reflect their specific needs and goals. In terms of ambitions, most of the MSs show commitment to biodiversity and natural resources management.

The CAP Plans show commitment to the Green Deal through various focused strategies. Bulgaria, Flanders, and Germany prioritize reducing greenhouse gas emissions, improving soil and water quality, and promoting renewable energy again setting specific targets. Wallonia and Latvia allocate significant portions of their “budgets to eco-schemes and environmental interventions”, reflecting their dedication to sustainable agricultural practices. Countries like Czechia, Estonia, and France emphasize “protecting water, soil, and biodiversity”, while also reducing emissions and enhancing carbon sequestration. Similarly, Italy, Croatia, and Lithuania focus on improving soil and water quality, promoting organic farming (Günther et al. 2024), (Lori et al. 2017) and implementing eco-schemes. Hungary, Austria, and Poland (Zieliński et al. 2024) aim to enhance biodiversity-friendly agriculture, sustainable nutrient management and implementing eco-schemes. Ireland, Greece, and Spain prioritize “climate change adaptation and biodiversity preservation”, with measures such as reducing chemical nitrogen usage and increasing tree planting. Lastly, Cyprus, Malta, Luxembourg, and the Netherlands focus on improving water and soil quality, reducing pesticide use(Finger and Möhring 2024), and increasing organic farming. In summary, the majority of MSs focus on Green Deal targets for the protection of soil, water and biodiversity.

**Fig. 11:**
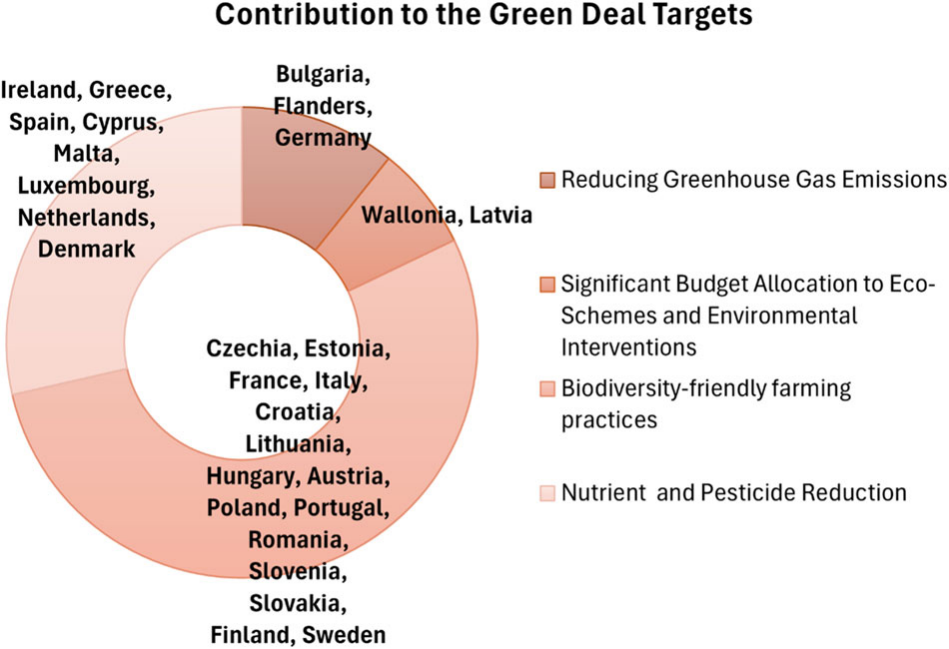
Greener CAP contribution to the Green Deal Targets priorities of MSs identified by the author

It’s worth to remark that the categories presented in Figs. 10 and 11 are closely related but serve distinct purposes. While Fig. 10 highlights the future environmental and climate ambitions of MSs, including their specific targets and strategic goals, Fig. 11 emphasizes the ongoing actions and contributions of MSs to the Green Deal priorities through their CAP Plans. For instance, both figures showcase themes such as reducing greenhouse gas emissions, biodiversity preservation, and sustainable water and soil management. However, Fig. 10 focuses on aspirational targets like carbon neutrality by 2050, whereas Fig. 11 represents tangible, budgeted actions like eco-schemes, organic farming promotion, and resource allocation for biodiversity-friendly agriculture.

**Fig. 12:**
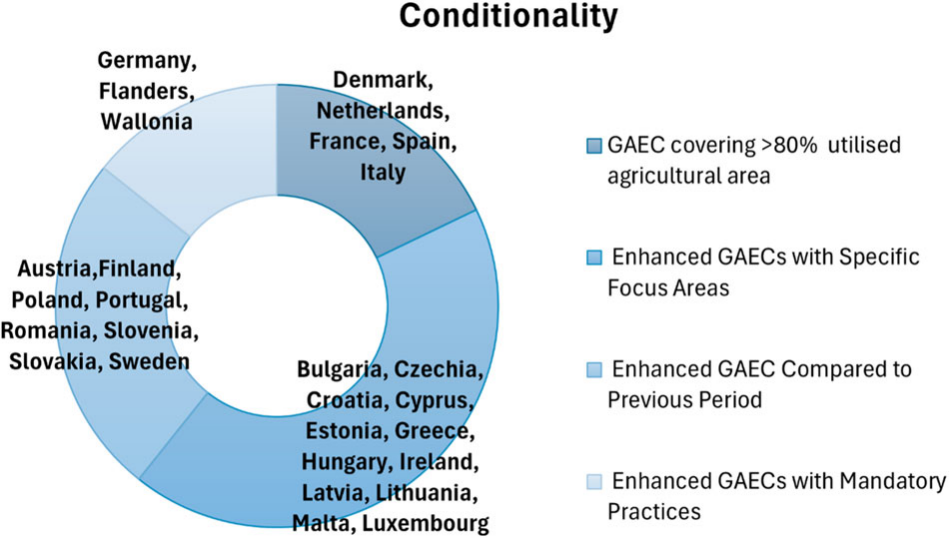
Greener CAP enhanced conditionality priorities of MSs identified by the author

In Fig. 12, Denmark, the Netherlands, France, Spain, and Italy have implemented enhanced standards covering a large portion of their agricultural areas, ensuring compliance with Good Agricultural and Environmental Conditions (GAECs)(Nikolina 2024). Countries like Bulgaria, Czechia, and Croatia have introduced “enhanced standards with specific focus areas”, such as eco-schemes and soil protection. Estonia, Greece, and Hungary have also implemented enhanced GAECs to protect soil and water quality, while Ireland(Osawe et al. 2024), Latvia and Lithuania have focused on protecting watercourses and improving soil conditions. Luxembourg ensures that almost all farms comply with GAECs, maintaining permanent grassland and ecological farmland. Austria, Poland, and Portugal have strengthened their GAECs compared to the previous period, demonstrating their commitment to continuous improvement. Germany, Flanders, and Wallonia have included mandatory climate and environmental practices, with incentives to maintain permanent grassland (Elliott et al. 2024) and support organic farming. In summary, farmers increasingly recognize the important role they play in safeguarding natural resources and achieving national environmental goals (McCormack et al. 2024).

**Fig. 13:**
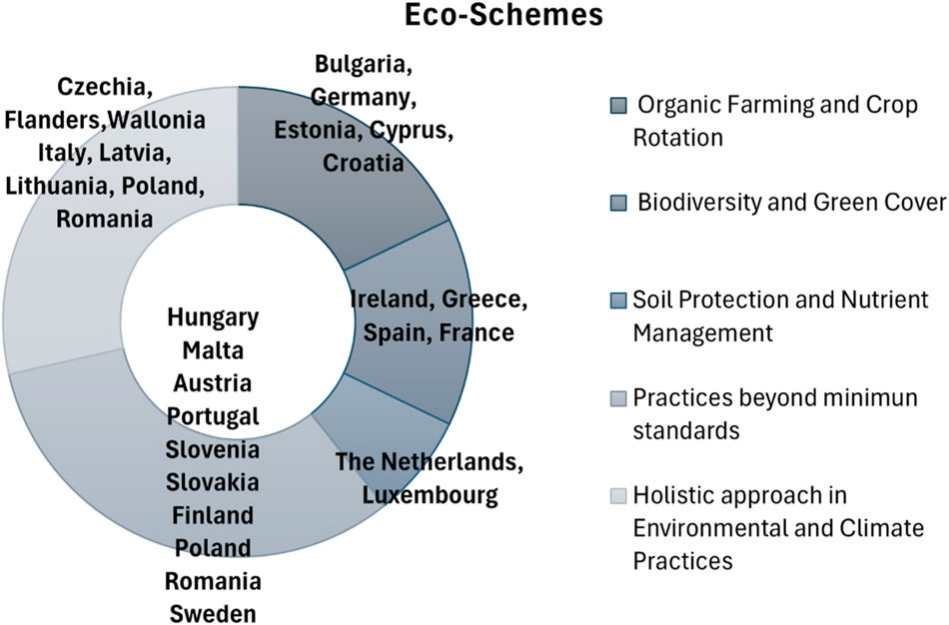
Greener CAP Eco-Schemes priorities of MSs identified by the author

Figure 13 shows a diverse range of practices aimed at enhancing environmental sustainability and climate resilience. Countries like Bulgaria, Germany, and Estonia focus on organic farming (Panday et al. 2024) and crop rotation (Costa et al. 2020), promoting healthier soil and reduced chemical use. Ireland, Greece, and Spain prioritize biodiversity and green cover (Capó-Bauçà et al. 2019), with practices aimed at improving climate, air, and water quality, and protecting wildlife. Soil protection and nutrient management are key in the Netherlands and Luxembourg, where efforts are made to reduce nutrient and pesticide use and establish buffer strips (Kumwimba et al. 2024), (Chen et al. 2024). Denmark, Hungary, and Malta implement practices that benefit the climate, environment, and animal welfare, often going beyond minimum legal requirements. General environmental and climate practices are widespread, with countries like Czechia, Flanders, and Wallonia incentivizing farmers to adopt environmentally friendly practices across their agricultural areas. So, climate and environmental benefits go larger across the MSs for the Eco-Schemes development.

**Fig. 14:**
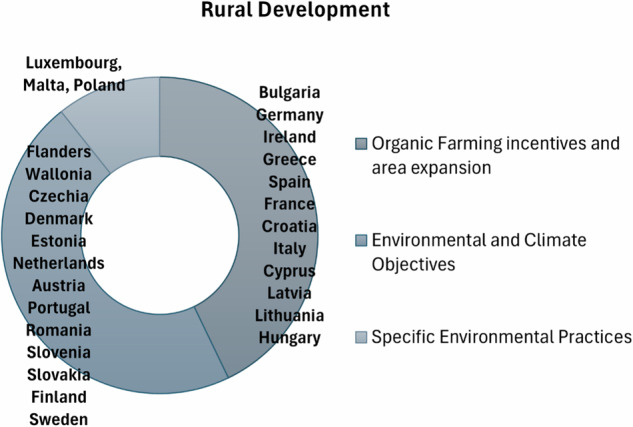
Greener CAP rural development priorities of MSs identified by the author

The MSs demonstrate a strong commitment to enhancing rural development (Fig. 14), with higher financial commitments and more ambitious targets than seen during the 2014–2020 period (European Commission: Directorate-General for Agriculture and Rural Development et al. 2024). Bulgaria, Germany, and Ireland have allocated significant financial resources to promote organic land cultivation, beekeeping (Prodanović et al. 2024), and livestock breeding. Germany, in particular, has dedicated nearly EUR 2.4 billion to organic farming and EUR 1.7 billion to biodiversity-related practices. Ireland aims to triple the area of agricultural land under organic production with an investment of EUR 256 million. Greece, Spain, and France have also committed substantial funds to environmental and climate objectives, including organic farming. Croatia and Italy have set ambitious targets to increase the area under organic farming, with Italy aiming for 25% by 2027. Countries like Cyprus, Latvia, and Lithuania prioritize agri-environmental schemes (Zindler et al. 2024a) and sustainable farming practices, with significant portions of their rural development budgets dedicated to these initiatives. Hungary aims to double the area under organic farming by 2027, allocating 38% of its rural development budget to agri-environmental interventions. Environmental and climate objectives are a key focus for Flanders, Wallonia, and Czechia, with substantial financial support for organic production. Denmark, Estonia, and the Netherlands have implemented initiatives to improve resource efficiency, reduce waste and emissions, and support biodiversity. Austria, Portugal, and Romania have allocated nearly 60% of their rural development budgets to environmental objectives, including area-based payments for environmentally (Zindler et al. 2024b) friendly practices. Luxembourg and Malta have introduced specific environmental practices, such as supporting less intensive livestock systems and investing in water storage and recycling. Poland focuses on soil protection and sustainable production methods.

**Fig. 15:**
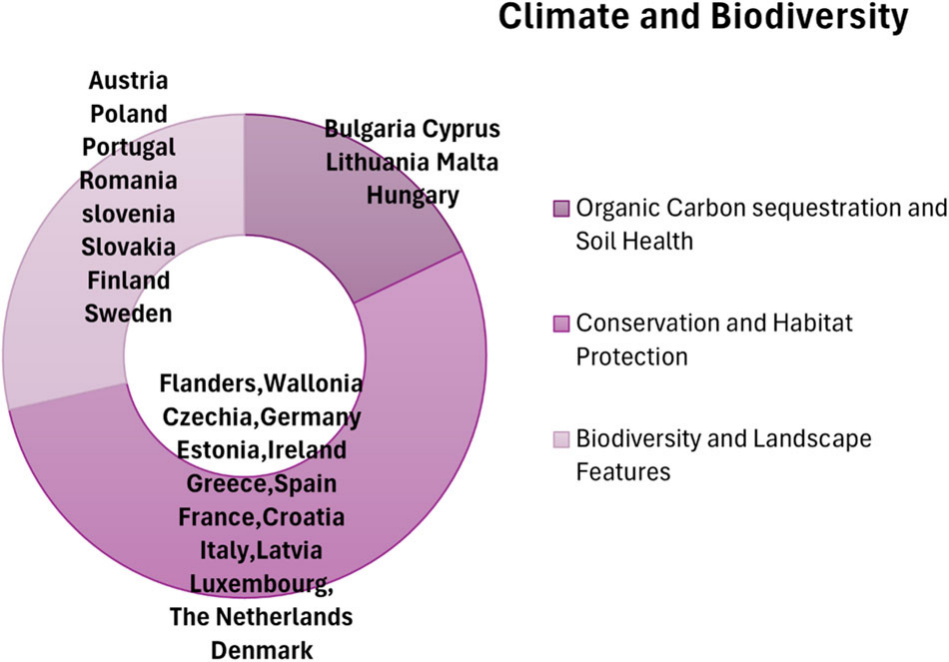
Greener CAP climate and biodiversity priorities of MSs identified by the author

The MSs exhibit a fair commitment to enhancing organic carbon and soil health, biodiversity (Mattalia 2024), and landscape features through various initiatives (Fig. 15). While the overarching objectives of improving soil conditions, increasing biodiversity, and promoting sustainable land use are shared, the methods and emphasis vary across countries based on their specific regional priorities. Bulgaria, Cyprus, and Lithuania focus on increasing organic carbon in soils (Blanco-Canqui et al. 2013), improving soil conditions, and supporting afforestation and forest regeneration (Breil et al. 2024). Malta and Hungary emphasize improving soil health, enhancing biodiversity, and supporting sustainable forest management. Countries like Flanders, Wallonia, and Czechia prioritize biodiversity and landscape features, with innovative commitments such as buffer strips, agroforestry systems, and sustainable meadow management (Buhk et al. 2018). Germany and Estonia focus on practices related to biodiversity, carbon sequestration, and soil and water quality improvements. Ireland, Greece, and Spain support organic farming, increasing agricultural land under organic farming, and enhancing biodiversity through financial aid and modern irrigation systems. France, Croatia, and Italy implement practices that enhance biodiversity, maintain landscape features, and reduce pesticide use. Latvia and Luxembourg focus on conserving and restoring biodiversity, improving animal welfare, and supporting non-productive surfaces and buffer strips. The Netherlands, Austria, and Poland aim to increase organic production and enhance biodiversity landscape features, including support for honey plants. Portugal, Romania, Slovenia, Slovakia, Finland, and Sweden promote renewable energy and sustainable agronomic practices alongside increasing organic production and enhancing biodiversity landscape features. Denmark focuses on conservation and habitat protection, improving and maintaining the conservation status of habitats and species in Natura 2000 protected areas, and committing to improving soil production potential. In summary, most of MSs invest in landscape and biodiversity conservation rather than reducing greenhouse gases.

To summarize, although many MSs pursue similar biodiversity and soil health objectives, the strategies they employ are often adapted to their specific environmental contexts. For example, buffer strips are a common tool across countries, yet their implementation varies depending on local landscape and water management needs. Likewise, while organic farming practices are encouraged throughout the EU, the specific focus areas such as crop types and regional biodiversity differ among nations.

## Discussion

The present study reveals a varied landscape of implementation levels across both NECPs and CAP Plans. At the state of the art, the extent of NEPCs and CAP Plans alignment with the NRL objectives varies, with some plans focusing more on specific environmental objectives while others already show a comprehensive framework that includes nature restoration. Moreover, while NECPs and CAP Plans can potentially complement each other and support the achievement of the goals of the NRL, several challenges need to be addressed.

Table 2 shows NECPs and CAP Plans cross-comparison obtained by the previous analysis and focuses on exploring overlap and synergies between the two plans and their potential alignment with the NRL targets.

**Table 2:** Potential areas of overlap and synergy between the NECPs and CAP Plans, with a focus on their alignment with the NRL targets

NRL Targets	NECPs Objectives	CAP Objectives	Potential Overlap & Synergy to NRL reporting
**Restoration targets based on existing legislation** (wetlands, forests, grasslands, rivers etc)	Enhancing carbon sinks through LULUCF Adaptation Renewable Energy	Eco-schemes to protect biodiverse habitats and sustainable land management	NECP actions remain insufficient in planning and implementation. CAP Plans manage habitats linked to agricultural areas more effectively. NRL reporting should leverage ongoing CAP efforts to coordinate with NECPs for measures beyond land use.
**Pollinating insects -** reversing decline by 2030	Reducing agricultural emissions and promoting biodiversity	Eco-schemes to reduce pesticide use and enhance crop diversity	NECPs and CAP Plans aim to reduce pesticide use and to protect biodiversity, indirectly concurring to improve habitats for pollinators. However, no explicit strategies for pollinators result in any MSs plans. This is a focal point that NRL reporting should address to reverse pollinators’ decline by 2030.
**Forest ecosystems** increase deadwood, uneven-aged forests, forest connectivity	Sustainable forest management and enhancing carbon stocks (commitment to LULUCF Regulation)	Conditionality on forest conservation practices	While most MSs’ NECPs include afforestation measures, they often lack specificity. Only some MSs take a clearer approach with robust CAP Plans incorporating agroforestry, targeted tree planting, and bird protection. NRL reporting should facilitate the convergence of ongoing NECP and CAP actions on forest ecosystems including more specific restoration targets.
**Urban ecosystems** no net loss of green space	Urban greening initiatives/measures	Support for urban-rural linkages and green infrastructure	In the NECPs, fewer than five MSs mention urban area greening, while in the CAP, the urban context is mentioned only when actions related to agriculture are implemented. This is certainly an area where there is little overlap between the NECPs and CAP Plans, and it needs to be further developed within the NRL reporting.
**Agricultural ecosystems** increase grassland butterflies, farmland birds, soil organic carbon	Reducing non-CO_2_ emissions and improving soil health	Eco-schemes for soil health and high-diversity landscape features	NECPs address non-CO_2_ emissions from agriculture, waste, and fugitive sources, though often with vague measures, while CAPs focus on soil health and diverse landscapes but include few targeted actions for butterfly conservation, highlighting a key area for NRL reporting.
**Marine ecosystems** restore seagrass beds, sediment bottoms	Coastal habitat protection and reducing marine pollution while addressing the impact of Renewable energy implementation	Sustainable management of agriculture in coastal and marine areas	NECPs prioritize coastal protection and pollution reduction but in the view of balancing marine renewable energy expansion. CAP Plans mention sustainable practices in coastal areas without specific marine restoration actions. NRL reporting should align with the EU Marine Strategy Framework Directive, which mandates pollution reduction, biodiversity restoration, and impact management in marine areas.
**River connectivity** remove barriers, restore 25,000 km of rivers	Enhancing river flow and rewilding initiatives	Sustainable water management practices in agriculture	NECPs address river basin management in some adaptation plans, balancing it with hydroelectric power, while CAP Plans more extensively cover freshwater management, offering a foundation for NRL reporting to support the restoration of 25,000 km of free-flowing rivers

In reviewing the potential overlap between NRL targets and existing measures in NECPs and CAP Plans for restoration targets based on existing legislation, it is evident that several habitats targeted by the NRL are already addressed under the other two policy frameworks. However, while the actions outlined in the NECPs are still insufficient in terms of detailed planning and implementation, the same habitats, particularly those involved in or adjacent to agricultural activities, are more thoroughly managed within the CAP plans. This suggests that NRL reporting should integrate existing efforts aimed at reversing habitat degradation already underway through CAP plans. Additionally, for measures that go beyond agriculture, there is a need for better coordination with what MSs have already declared in their NECPs.

The NRL target to reverse the decline of pollinating insects by 2030 aligns with broader objectives in both NECPs and CAP Plans. CAP eco-schemes emphasize reducing pesticide use and enhancing crop diversity, which can improve habitats for pollinators. However, despite these overlaps, neither the NECPs nor the CAP Plans from any MS explicitly include targeted strategies for pollinators’ restoration, indicating a focal point that needs to be addressed in NRL reporting.

Although NECPs of most MSs include measures for afforestation (under LULUCF commitment) to support climate adaptation and mitigation through the forest ecosystem, these actions are often vague and lack specificity. Some MSs have more robust approaches, incorporating measures like agroforestry, targeted tree planting and bird protection under their CAP Plans to enhance both carbon sequestration and ecosystem resilience. As for the Restoration targets based on existing legislation, NRL reporting should integrate existing efforts in CAP and NECPs integrating its specific targets (like increasing forests’ connectivity, etc.).

Regarding urban ecosystems, in the NECPs, fewer than five MSs mention urban area greening, while in the CAP, the urban context is mentioned only when actions related to agriculture are implemented. This is certainly an area where there is little overlap between the NECPs and CAP Plans, and it needs to be further developed within the NRL reporting.

For agricultural ecosystems, NECPs might focus on reducing non-CO_2_ emissions (methane, nitrous oxide and fluorinated gases) from agriculture, but also from waste, and fugitive sources. As already mentioned for other targets, NEPCs are often vague and lack specific measures. CAPs, in turn, are largely centred on agricultural ecosystems, promoting eco-schemes that enhance soil health and maintain diverse landscapes. However, any NEPCs and just a few CAP Plans currently include targeted measures for butterfly conservation, which leaves a crucial area for NRL reporting.

For marine ecosystems, NECPs aim to protect coastal habitats and reduce marine pollution, but mainly in the view to balancing these goals with the expansion of renewable energy installations in marine environments. CAP Plans, meanwhile, support sustainable rural coastal areas, but don’t address specific actions to restore marine ecosystems. NRL reporting should align with the Marine Strategy Framework Directive (MSFD) (European Parliament and Council of the European Union 2008) which refers to MSs obligation to implement programs to reduce pollution, restore marine biodiversity, and protect vulnerable marine areas. They are also required to monitor and report on the status of marine waters and take action to reduce the impact of human activities, including fishing and pollution (Perissi and Bardi 2021).

On river connectivity, NECPs mention river basin management in some adaptation plans, where, similar to renewable energy in marine ecosystems, it is balanced with hydroelectric power expansions. CAP Plans discuss water management more extensively, covering rivers, lakes, and other freshwater resources. In this respect, CAP Plans already provide an initial source of information for structuring NRL reporting aimed at achieving the restoration target of 25,000 km of free-flowing rivers.

Beyond the focused contribution to NRL targets, the analysis of NECPs and CAP Plans reveals other areas of overlap and synergy that should not be at the centre of the NRL reporting but need to be considered to jointly shape the three reporting plans in the broader context of ecosystem restoration (Tedesco et al. 2023). First, in terms of decarbonization by land use, both plans emphasize reducing greenhouse gas emissions by LULUCF and increasing soil carbon uptake via sustainable agriculture. NECPs promote nature-based solutions to improve ecosystem resilience, and CAP plans prioritize the protection of high-diversity landscapes. However, coordinated implementation remains essential to avoid redundant or conflicting efforts in biodiversity conservation.

Renewable energy and land use goals also intersect, as NECPs encourage expanding renewable infrastructure (such as bioenergy and solar farms, wind energy off shore and in shore, and hydroelectric production), while CAP Plans stress protecting agricultural land from unmanaged renewable expansion. This balance is crucial, given the potential conflict between bioenergy projects and CAP’s biodiversity objectives, highlighting the importance of aligning renewable energy targets with land conservation practices. Additionally, both NECPs and CAP Plans aim to improve water management and soil health, with NECPs focusing on water efficiency and climate resilience in water resources, while CAP Plans emphasize soil health enhancement and nutrient runoff reduction. The shared promotion of practices like buffer strips and nutrient management suggests considerable overlap in water and soil quality protection measures. Finally, regarding climate adaptation, NECPs and CAP Plans advocate for increasing resilience to climate change through adaptation strategies and by enhancing the robustness of agricultural systems. These aligned goals underscore the potential of NECPs and CAP Plans to advance environmental resilience and sustainability if carefully coordinated jointly (Stuch and Alcamo 2024), (Mondière et al. 2024).

In interpreting the CAP Plans, overlapping categories were identified as a reflection of the multifaceted approaches adopted by MSs to achieve “Greener CAP” objectives. This overlap underscores the complexity of aligning national priorities with EU-level targets. While thematic grouping facilitated comparative analysis, future work could develop more standardized methods for assessing the effectiveness of CAP measures in delivering environmental benefits. Such standardization would enhance comparability across MSs, helping to identify best practices and gaps more effectively. Moreover, while NECPs and CAP Plans often outline clear objectives, they frequently lack detailed roadmaps or actionable strategies to achieve them. This discrepancy raises questions about the realism of current plans and their ability to meet NRL goals within the required timeframe.

The study also points out the shortage of research and comprehensive tools necessary for developing national plans (NEPCs, CAP Plans, and NRL Plans) that can effectively promote transparency and coordination among them. This is essential to avoid wasting valuable financial resources and time, especially given the urgency of restoring environmental integrity. In this regard, the recent work by Phoa (Phoa 2024) reports the CAP Plans have shifted from a centralized framework to a more devolved one, now granting MSs higher flexibility in their implementation. However, compliance with EU-wide social and environmental plans, such as the Green Deal and the NRL, might limit this flexibility. Therefore, it might be necessary to explore whether the comprehensive tools could be further decentralized, and determine the extent to which they could enhance the performance of MSs in achieving their objectives.

As final remarks, the author outlined the following recommendations to improve synergies between policymakers and researchers (Zaki and Dupont 2024) to guide future steps to meet restoration objectives :

- Recommendations for Policymakers:
  1. Cross-Plan Integration. Many NECPs lack specificity in adaptation measures like afforestation and biodiversity restoration: policymakers should work to align with efforts already under CAP Plans, incorporating in NEPCs clearer, measurable targets, such as specific agroforestry habitat preservation measures, to complement CAP Plans Eco-schemes.
  2. Address Gaps in Urban Ecosystem Restoration. Since urban greening efforts are scarcely mentioned in both NECPs and CAP Plans, policymakers should prioritize the development of MSs urban ecosystem restoration framework within the NRL reporting. This would facilitate consistent reporting and implementation of urban greening measures that go beyond agricultural contexts.
  3. Coordination on Renewable Energy Impacts. Policymakers need to recognise the conflict between renewable energy expansion and biodiversity goals and establish guidelines that integrate CAP’s land conservation with NECP renewable energy targets. This balance would ensure that bioenergy projects and other renewables support, rather than undermine, biodiversity and conservation goals.
- Recommendations for Researchers:
  1. Advanced Assessment Tools for Renewable Energy Expansion. New tools should evaluate how renewable energy expansion impacts land-use strategies and carbon sinks, addressing ecological and carbon storage objectives. These tools would enable policymakers to balance NECP renewable energy goals with land use, including compatibility with agriculture practices and conservation priorities, ensuring that renewable projects contribute positively to land and carbon management (Koponen et al. 2024).
  2. Targeted Actions for Urban and Marine Ecosystems. Researchers should identify strategies related to urban greening and marine ecosystem protection and develop predictive models to accomplish NRL targets in these areas. This would provide a data-driven foundation to advocate for specific green urban areas and marine restoration efforts within future NECPs and CAPs.

Finally, the author recognises that rather than providing an exhaustive breakdown of each specific practice and mechanism, this analysis focuses on the broad diversity of environmental and climate objectives across MSs within the NEPCs and CAP frameworks. Future works could benefit from this benchmark to achieve a more systematic classification of the strategies to better quantify the differences in MSs’ approaches toward NRL implementation, for instance breaking down the study into smaller topics.

## Conclusion

The study’s findings underscore a critical challenge in the EU’s approach to achieving its ambitious climate and environmental goals: while MSs have made progress in setting targets and expressing commitments, a substantial gap remains in the implementation of effective and robust measures to achieve these objectives. This discrepancy is particularly evident in the areas of land use, agriculture, and the integration of renewable energy with land-use strategies, where the analysis reveals a lack of detailed, actionable plans across many MSs. The insufficient analytical basis for policy implementation further weakens the credibility of these plans, raising concerns about the EU’s ability to meet its 2030 and 2050 climate goals. The study also highlights varying levels of alignment between NEPCs and CAP Plans actions and the NRL targets. Some MSs’ plans show a comprehensive framework that includes nature restoration, yet many others only address individual objectives without fully integrating them. There is general recognition of the importance of nature-based solutions and adaptation strategies, but these are often underdeveloped, with limited resource allocation and inadequate quantification of impacts. Key NRL targets, such as reversing the decline of pollinators, are inconsistently addressed, with both NECPs and CAP Plans lacking targeted strategies for pollinator restoration. Moreover, measures for marine ecosystems, forest connectivity, and urban greening remain vague or omitted.

Given these findings, there is an urgent need for cross-plan integration to improve transparency, coordination, and resource efficiency. While the NRL already requires alignment in the national restoration plans, adjustments are needed in the other plans, such as NECPs and CAP Plans, to ensure they fully support the overarching restoration objectives. The study recommends that policymakers incorporate clear, targeted adaptation measures and habitat preservation efforts within NECPs to complement CAP eco-schemes, close gaps in urban ecosystem restoration, and establish balanced guidelines that align CAP’s land conservation priorities with NECP renewable energy targets and overarching restoration objectives.

## Supplementary information

Supplementary material_NRL_CAPplans

Supplementary material_NRL_NEPCs

## Data Availability

Data is provided within the manuscript with supplementary information files
